# Obacunone Attenuates Liver Fibrosis with Enhancing Anti-Oxidant Effects of GPx-4 and Inhibition of EMT

**DOI:** 10.3390/molecules26020318

**Published:** 2021-01-09

**Authors:** Yongquan Bai, Wenwen Wang, Li Wang, Lirong Ma, Dongsheng Zhai, Furong Wang, Rui Shi, Chaoyang Liu, Qing Xu, Guo Chen, Zifan Lu

**Affiliations:** 1 The College of Life Sciences, Northwest University, Xi’an 710127, China; Byqyongquan@163.com (Y.B.); m920659372@163.com (L.M.); wangfr1216@163.com (F.W.); myruio0405@163.com (R.S.); m13892081758@163.com (C.L.); 3025054@163.com (Q.X.); 2 State Key Laboratory of Cancer Biology, Department of Biopharmaceutics, Air Force Military Medical University, Xi’an 710083, China; kjjydwangww@163.com (W.W.); wanglilaura@163.com (L.W.); 3 Department of Pharmacology, School of Pharmacy, Air Force Medical University, Xi’an 710083, China; zhaidongsheng.ok@163.com

**Keywords:** obacunone, liver fibrosis, EMT, hepatic stellate cells, GPx-4, TGF-β, oxidative stress

## Abstract

Obacunone, a limonin triterpenoid extracted from Phellodendronchinense Schneid or Dictamnus dasycarpusb Turcz plant, elicits a variety of pharmacological effects such as anti-inflammatory, anti-neoplastic, anti-oxidation, and anti-lung-fibrosis ones. However, the anti-fibrotic effect of obacunone and the detailed underlying mechanism in liver fibrosis remain unclear. Liver fibrosis is a debilitating disease threatening human health. Transforming growth factor (TGF)-β/P-Smad is a major pathway of fibrosis featured with epithelia mesenchymal transformations (EMT) and collagen depositions, accompanying with excessive oxygen-free radicals. Nrf-2 acts as a key anti-oxidative regulator driving the expressions of various antioxidant-related genes. Glutathionperoxidase-4 (GPx-4) is a member of the glutathione peroxidase family that directly inhibits phospholipid oxidation to alleviate oxidative stress. In the present study, we aimed to explore the role of obacunone in mouse liver fibrosis model induced by carbon tetrachloride (CCl4) and in hepatic stellate cells (LX2 cell line) challenging with TGF-β. Obacunone demonstrated potent ameliorative effects on liver fibrosis both in activated LX2 and in mice liver tissues with reduced levels of α-SMA, collagen1, and vimentin. Obacunone also remarkably suppressed the TGF-β/P-Smad signals and EMT process. Meanwhile, obacunone exerted a potent anti-oxidation effect by reducing the levels of reactive oxygen species (ROS) in both models. The antioxidant effect of obacunone was attributed to the activation of GPx-4 and Nrf-2. In addition, the therapeutic effect of obacunone on LX2 cells was significantly removed in vitro plus with GPx-4 antagonist RSL3, in parallel with the re-elevated levels of ROS. Thus, we demonstrate that obacunone is able to attenuate liver fibrosis via enhancing GPx-4 signal and inhibition of the TGF-β/P-Smad pathway and EMT process.

## 1. Introduction

Chronic hepatitis is usually caused by virus hepatitis, nonalcoholic steatitis, alcoholic hepatitis, and autoimmune hepatitis. Persistent hepatic injury may end up in liver fibrosis, or even liver cancer, which fatally affected human health [1]. Liver fibrosis is a type of wound non-healing response under the continuous injury factors characterized by the exaggerated accumulation of extracellular matrix (ECM) and scar formation [2]. Hepatic stellate cells (HSCs) are usually in a rest state under normal conditions, they became aberrantly activated under chronic liver injury and featured with high proliferation and secretions of ECM [3], denoted as myofibroblasts [4]. Understanding the mechanism of HSCs activation is of great value for the development of novel therapeutics for treating hepatic fibrosis.

Transforming growth factor (TGF-β) promotes inflammatory responses by stimulating the expression of Interleukin-1 (IL-1) and Interleukin-6 (IL-6) in monocytes [5]. Moreover, it induces cancer metastasis via epithelial mesenchymal transformation (EMT) and tumor immune escaping [6,7,8]. EMT refers to a dynamic physiological process wherein epithelial cells lose their polarity and transform into mesenchymal cells such as fibroblasts and myofibroblasts, thus leading to the development of fibrosis [9]. The occurrence of EMT is always associated with the downregulation of E-cadherin and the upregulation of mesenchymal cell’s markers, such as N-cadherin and snai1 [10]. TGF-β-Smad activation is one of the main intracellular pathways in EMT as well as myofibroblast formation [11].

Oxidative stress is considered as one of the primary causes of fibrosis with overproductions of reactive oxygen species (ROS) [12]. The high level of ROS causes structural damage to biomolecules, such as protein, lipids, and DNA, eventually leading to cell injury and the promotion of fibrosis [13]. Under oxidative stress, the dimer of Nrf-2-Keap-1 is dissociated, nuclear respiratory factor-2 (Nrf-2) is transported into nucleus, and induced the expression of various antioxidant enzymes by binding with the antioxidant response elements (ARE) to reduce the levels of ROS [14]. Glutathione peroxidase-4 (GPx-4) acts as an antioxidant protein with glutathione peroxidase activity, being mainly responsible for the inhibition of phospholipid oxidation and ROS production in ferroptosis process [15]. Mice studies have shown that fibrosis is aggravated after loss of either Nrf-2 or GPx-4 [16,17].

Presently, there are several drugs available for the treatment of liver fibrosis, including those with the properties of anti-inflammation [18], antioxidant [19], and peroxisome proliferators-activated receptors (PPAR) agonist [20]. In a recent study, Prof. Yu found that patients with liver cirrhosis have higher lipid oxidation levels in comparison to the healthy controls in clinical specimens [21]. Several antioxidant drugs such as resveratrol [19] and ursolic acid [13] have demonstrated good effects on fibrosis. This evidence cumulatively suggests that anti-oxidation is a promising strategy for treating anti-fibrosis.

Pirfenidone (PFD) is a pyridinone drug for idiopathic pulmonary fibrosis (IPF), while it also manifests a good therapeutic effect on liver fibrosis in multiple studies [22,23]. Thus, PFD is selected as a positive control medicine in this study. Obacunone (Oba) is a natural limonoid compound extracted from rutaceous plants like Phellodendri and Dictamnusdasycarpus [24], which possess various biological properties such as anti-inflammation [25], antioxidation [26], and anti-tumor ones [27]. Recent reports have revealed that Oba is a strong Nrf-2 agonist, which effectively alleviated lung fibrosis by CCL4 induction and oxidative damage to MDA-MB-231 cells caused by H_2_O_2_. More importantly, Oba is non-toxic to mice [28]. However, no reports on the effect of Oba in liver fibrosis are reported, especially on GPx-4 mediating anti-phospholipid oxidation, TGF-β-Smad signals, and EMT process. Herein, we intend to explore the effects of Oba on liver fibrosis and define the underlying molecular mechanisms.

## 2. Results

### 2.1. Oba Inhibited TGF-β-Induced Lx-2 Activation

**Figure 1:**
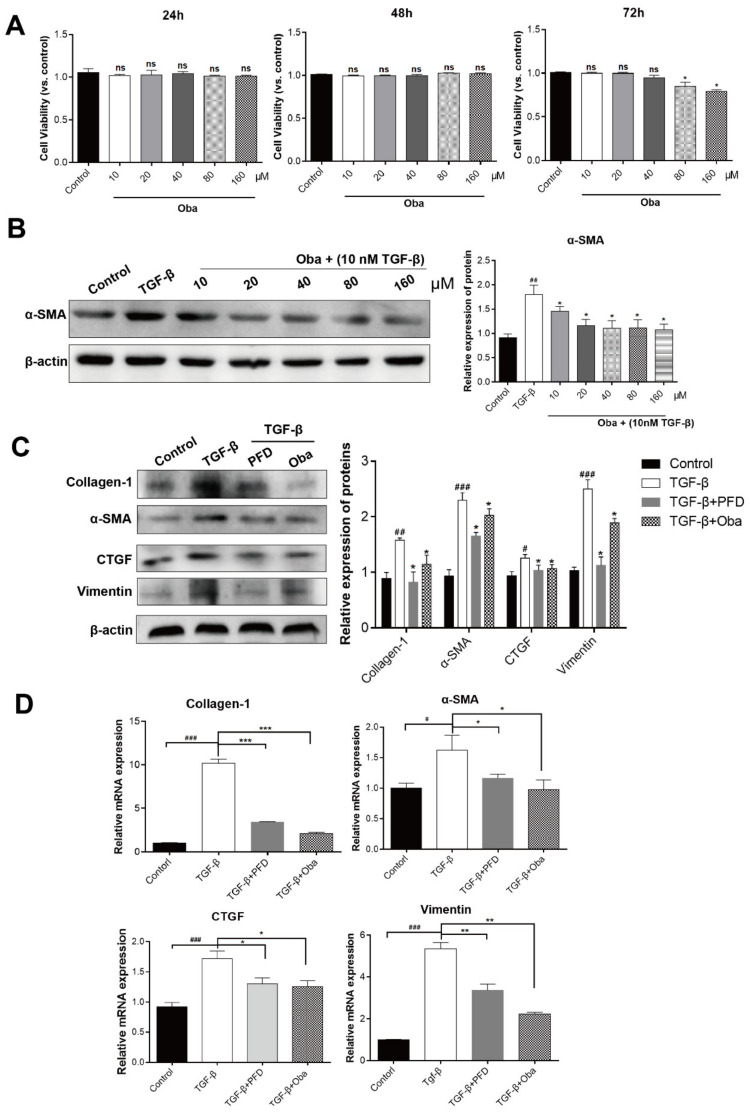

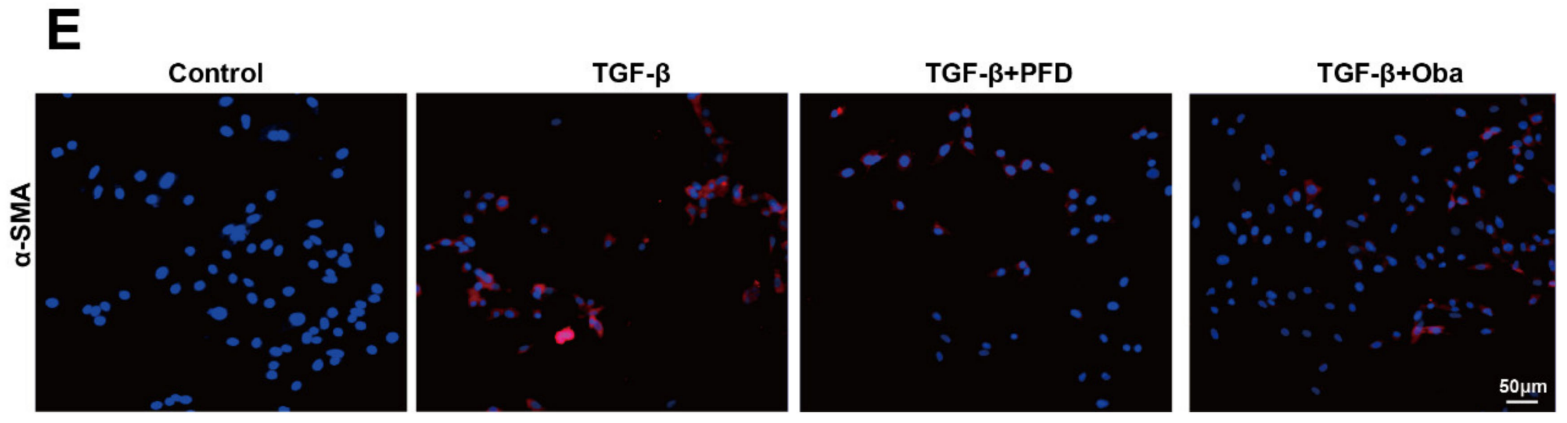
Obacunone (Oba) inhibited transforming growth factor (TGF)-β-induced Lx-2 activation. (**A**) The effects of Oba (0–160 μM, 24 h/48 h/72 h) on the Lx-2 cell viability as determined by the Cell Counting Kit-8 (CCK8) kit in accordance with the manufacturer’s protocol. (**B**) Quantification of the α-SMA protein expression as assessed by Western blotting for the TGF-β-induced Lx-2 cells activation after treatment with different concentrations of Oba (0–160 μM). (**C**) Quantification of collagen-1, vimentin, α-SMA, and CTGF protein expression as assessed by Western blotting in vitro. (**D**) Quantification of collagen-1, vimentin, and α-SMA mRNA expression as assessed by real-time qPCR. (**E**) Immunofluorescence staining for α-SMA (red) in LX-2 cells. The cell nucleus was counter-stained with DAPI (blue). The results were expressed as means ±SEM (*n* ≥ 3). *ns (italic)*, ^#^, ^##^, ^###^ indicate *p* > 0.05, *p* < 0.05, *p* < 0.01, *p* < 0.001 compare with control group; ns, *, **, *** indicate *p* > 0.05, *p* < 0.05, *p* < 0.01, *p* < 0.001 compare with model group.

Since the activated HSCs (aHSCs) played a critical role in liver fibrosis [3], we firstly observed the effects of Oba on liver fibrosis in aHSCs. In this experiment, we used the Cell Counting Kit-8 (CCK8) to detect toxicity of Oba on LX-2 cells, a type of human HSCs line. We found that LX-2 cells survival was not significantly affected by Oba at the concentration range of 10 to 160 μM during 24/48/72 h treatment (Figure 1A). Then, the LX-2 cells were stimulated with 10 ng/mL TGF-β for 24 h and then treated with Oba or not. the protein expression of α-Smooth muscle actin (α-SMA) was effectively inhibited at concentrations of >20 μM Oba. Interestingly, with the increased concentration of Oba, the α-SMA inhibition effect did not increase significantly (Figure 1B). Thus, we selected 20 μM concentration for the following experiment, with PFD serving as the positive control. Oba effectively inhibited the protein levels of α-SMA, collagen-1, vimentin, and connective tissue growth factor (CTGF) (Figure 1C), in aHSCs [3,4]. In addition, Oba reduced the mRNA expression levels of α-SMA, collagen-1, and vimentin (Figure 1D). The immunofluorescence results further verified that the positive staining signals of α-SMA were obviously reduced by Oba (relative to that in the TGF-β group, as red cell count reduced after treatment with Oba) in Lx-2 (Figure 1E). These results suggest that Oba exerts a good inhibition effect on the Lx-2 activation.

### 2.2. Oba Inhibited TGF-β/Smad Signals, EMT Process and Exerted Anti-Inflammatory Effect

**Figure 2:**
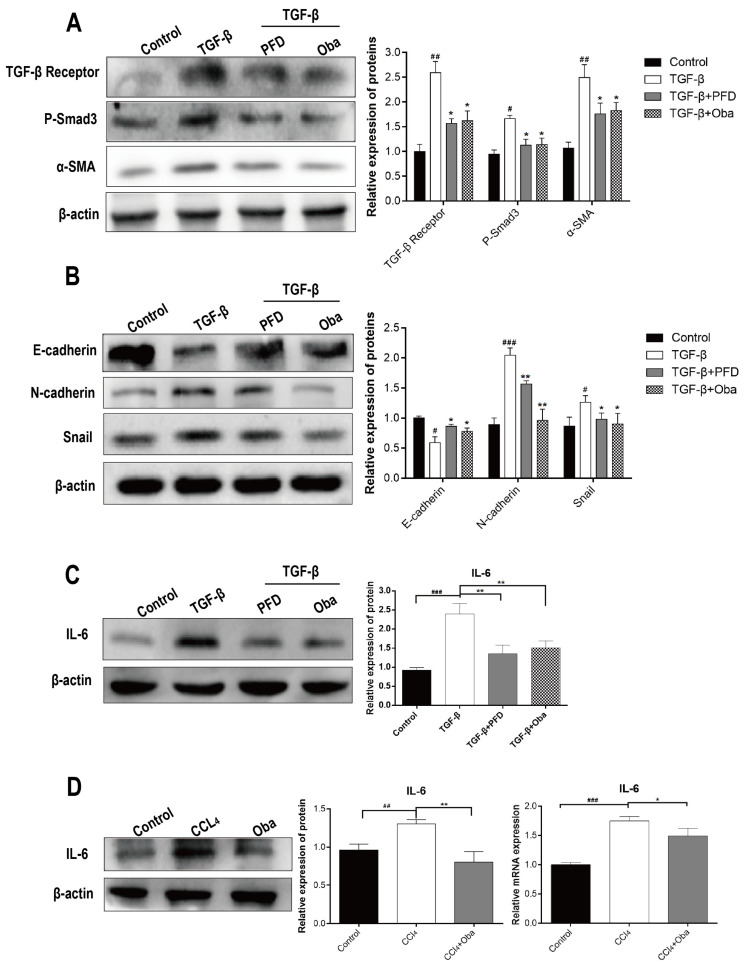
Oba exposure inhibited the TGF-β/SMAD/SMA signaling pathway and epithelia mesenchymal transformations (EMT) and showed anti-inflammatory effect. (**A**) Quantification of the TGF-β/Smad signaling pathway protein expression was assessed by Western blotting in vitro. (**B**) Quantification of EMT-associated protein expression as assessed by Western blotting in vitro. (**C**) Quantification of the IL-6 protein expression as assessed by Western blotting in vitro. (**D**) Quantification of the IL-6 protein and mRNA expressions as assessed by Western blotting and real-time qPCR in 20-nM CCl4-induced inflammation of Hegp-2 cells after treatment with 20-μM Oba. The results were expressed as means ±SEM (*n* ≥ 3). ^#^, ^##^, ^###^ indicate *p* < 0.05, *p* < 0.01, *p* < 0.001 compare with control group; ns, *, ** indicate *p* > 0.05, *p* < 0.05, *p* < 0.01 compare with model group.

To define the molecular mechanisms underlying the above anti-fibrosis effects, the protein expression levels of TGF-Receptor, P-Smad, and α-SMA were observed, which were significantly inhibited by Oba in aHSCs (Figure 2A). In addition, the protein expression levels of N-cadherin and snail were reduced, while the protein expression level of E-cadherin was promoted (Figure 2B), indicating that the EMT pathway was blocked too [3]. In addition, we found that Oba induced a reduced level of IL-6 (Figure 2C). To further evaluate the effect of Oba on inflammation, we first stimulated the HepG-2 cells (a type of human hepatocellular carcinoma cell line) with 20 nmol CCl4, followed by the treatment with 20 μM Oba. These results showed that Oba could reduce the mRNA and protein expression levels of IL-6 in CCl4 -stimulated HepG-2 (Figure 2D). On the whole, oba displayed multiple anti-fibrosis effects by inhibiting TGF-β/Smad signals, EMT process, as well as anti-inflammatory.

### 2.3. Anti-Fibrosis Effects of Oba Was Dependent on the Enhanced Antioxidant Capacity by Activating GPx-4

**Figure 3:**
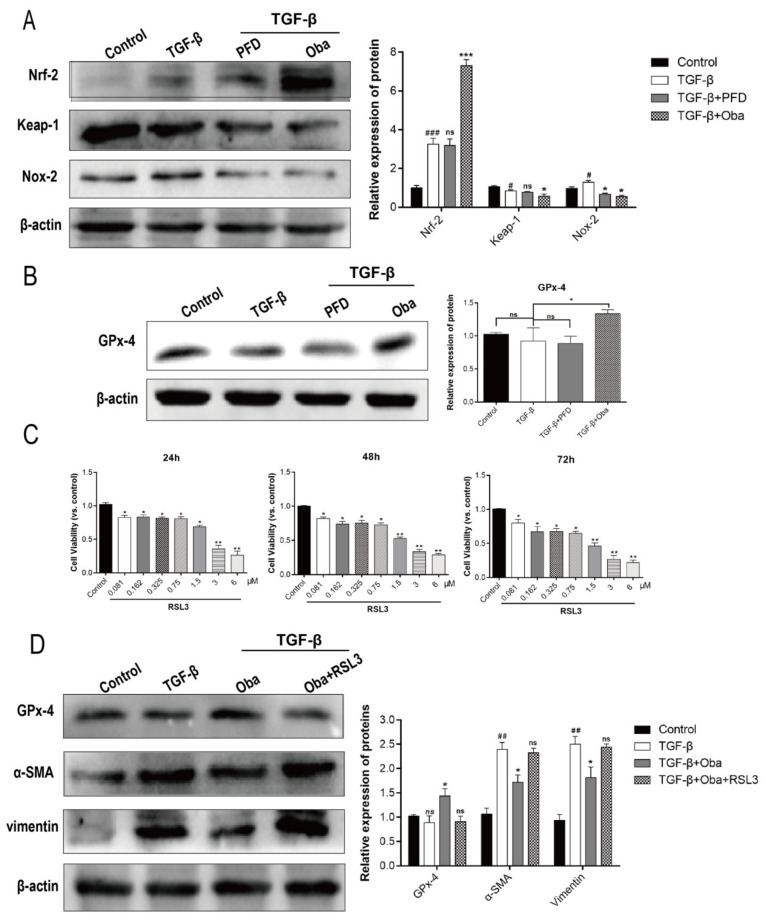

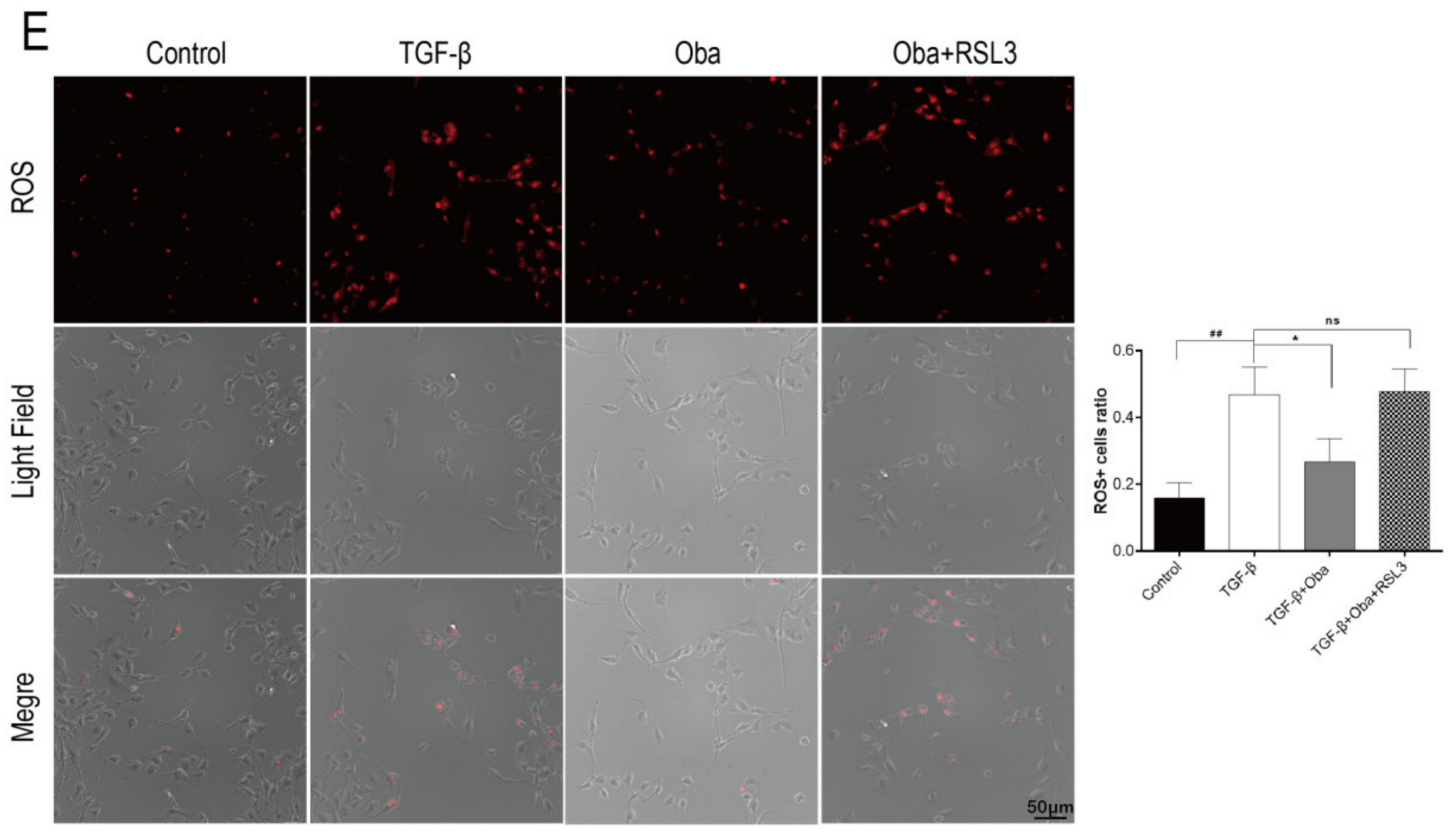
The anti-fibrosis effects of Oba were mediated by activated GPx-4-induced antioxidant capacity by reducing phospholipid oxidation. (**A**) Quantification of Nrf-2, Keap-1, and Nox-2 protein expressions as assessed by Western blotting in vitro. (**B**) Quantification of the GPx-4 protein expression as assessed by Western blotting in vitro. (**C**) The effects of RLS3 (0–6 μM, 24 h/48 h/72 h) on the Lx-2 cell viability as determined by the CCK8 kit in accordance with the manufacturer’s protocol. (**D**) Quantification of GPx-4, α-SMA, and collagen-1 protein expression as assessed by Western blotting with the interference of RSL3 in vitro. (**E**) Reactive oxygen species (ROS) (red) staining by Dihydroethidium (DHE). Cell morphology was observed under bright-field microscopy. The results were expressed as means ± SEM (*n* ≥ 3). *ns* (italic), ^#^, ^##^, ^###^ indicate *p* > 0.05, *p* < 0.05, *p* < 0.01, *p* < 0.001 compare with control group; ns, *, **, *** indicate *p* > 0.05, *p* < 0.05, *p* < 0.01, *p* < 0.001 compared with model group.

In order to further define the underlying molecular mechanisms of Oba in aHSCs, we discovered that Oba activated Nrf-2/Keap-1 pathway and reduced the protein expressions of Nox-2 (Figure 3A). In addition, we found that Oba increased the protein expression level of GPx-4 (Figure 3B). To validate the importance of GPx-4-mediated signal, RSL3, which is an inhibitor of GPx-4 [29], was selected to deal with Lx-2. Examination by using the CCK8 (Figure 3C) revealed that, at concentrations of RSL3 > 0.75 μM, the cell viability of Lx-2 was significantly decreased; therefore, 0.75-μM RLS was selected to intervene in the expression of GPx-4. The Western blot results showed that when RSL3 was added in aHSCs, GPx-4 protein expression was reduced and the profibrotic markers including α-SMA and vimentin were all reversed significantly, indicating the importance of GPx-4 in mediating the anti-fibrosis effects of Oba (Figure 3D). Meanwhile, Oba obviously reduced the ROS level of aHSCs by ROS fluorescent detectors, and this effect was apparently blocked by RSL3 (Figure 3E). These data indicated that the lower phospholipid ROS level mediated by GPx-4 was the main anti-fibrosis signals of Oba.

### 2.4. Oba Attenuates CCl4-Induced Mouse Liver Fibrosis

**Figure 4:**
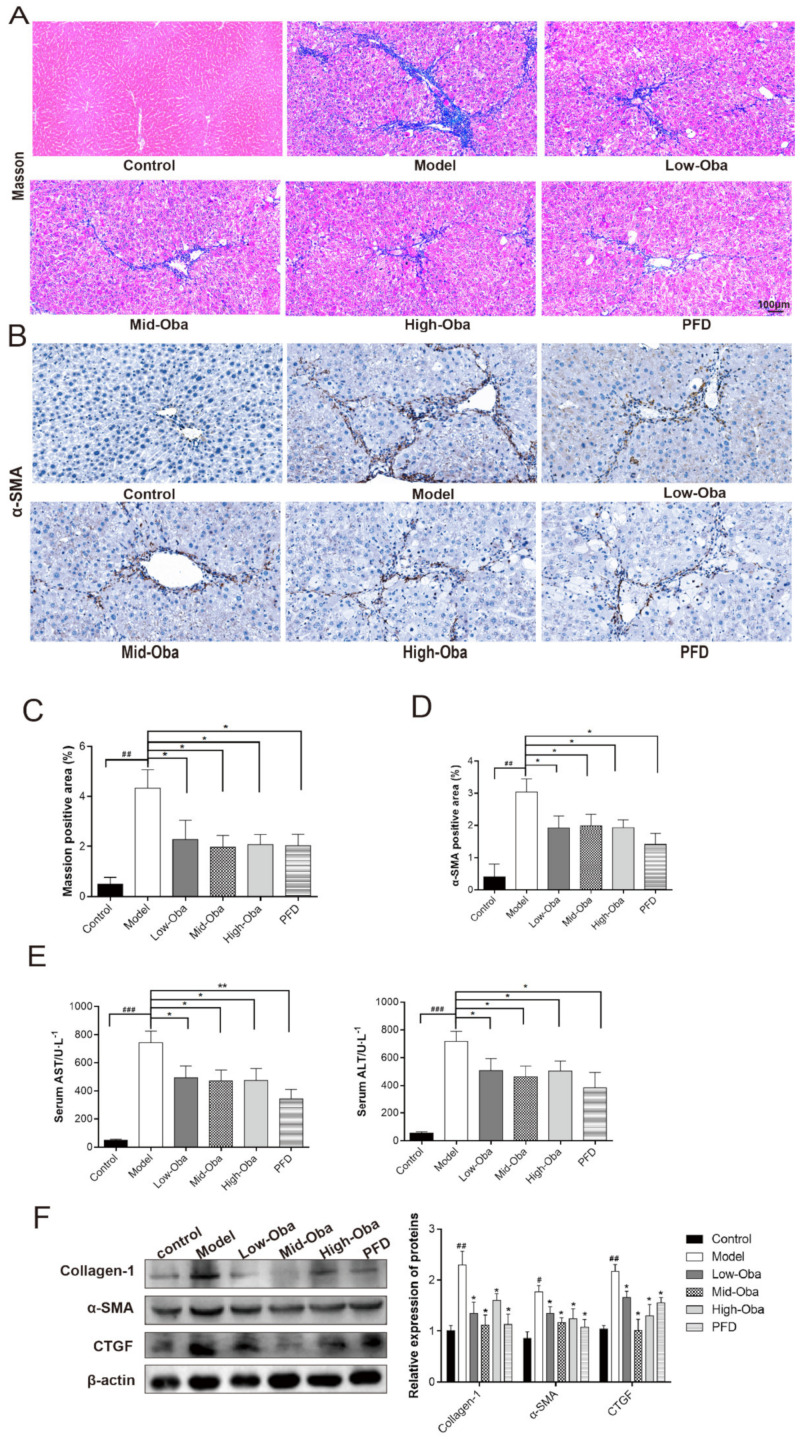
Oba attenuates CCl4-induced mouse liver fibrosis. (**A**) Masson staining of liver. (**B**) Immunohistologic staining for α-SMA (brown) in liver tissue. (**C**) Statistics of Masson positive areas. (**D**) Statistics of α-SMA positive areas. (**E**) Levels of serum aspartate transaminase (AST) and alanine transaminase (ALT). (**F**) Quantifications of Collagen-1, CTGF, α-SMA protein expression was assessed by Western blots from the mice in-vivo models. The results were expressed as means ±SEM (*n* ≥ 3). ^#^, ^##^, ^###^ indicate *p* < 0.05, *p* < 0.01, *p* < 0.001 compare with control group; ns, *, ** indicate *p* > 0.05, *p* < 0.05, *p* < 0.01 compare with model group.

CCl4-induced liver fibrosis is one of the most commonly used mouse models to study liver fibrosis [3]. At histological level, Oba significantly alleviated the liver pathology, evidenced by the ameliorated collagen depositions indicated with Masson staining and anti-SMA staining. (Figure 4A–D). Then, serum levels of alanine transaminase (ALT)/aspartate transaminase (AST) were determined to test the damage levels of liver, compared to the CCL4 mice, the low dose Oba group and PFD group significantly decreased the ALT levels (*p* < 0.05) (Figure 4E). Expectedly, Oba inhibited the protein expression levels of collagen-1, CTGF, and α-SMA in mouse fibrotic livers, which were consistent with the immunohistologic results (Figure 4F). These in-vivo results illustrated that Oba significantly improved the CCl4-induced mouse liver fibrosis.

### 2.5. Oba Enhanced the Expression of GPx-4, Decreased Inflammation, EMT-Related Protein Expressions and Reduced the Lipid Oxidation Levels in CCl4-Induced Liver Fibrosis

**Figure 5:**
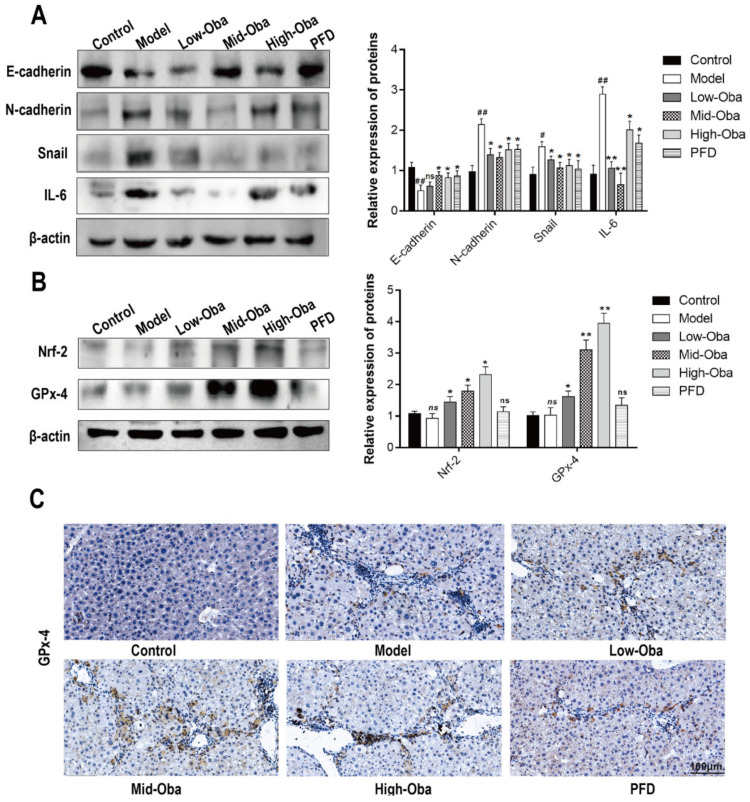

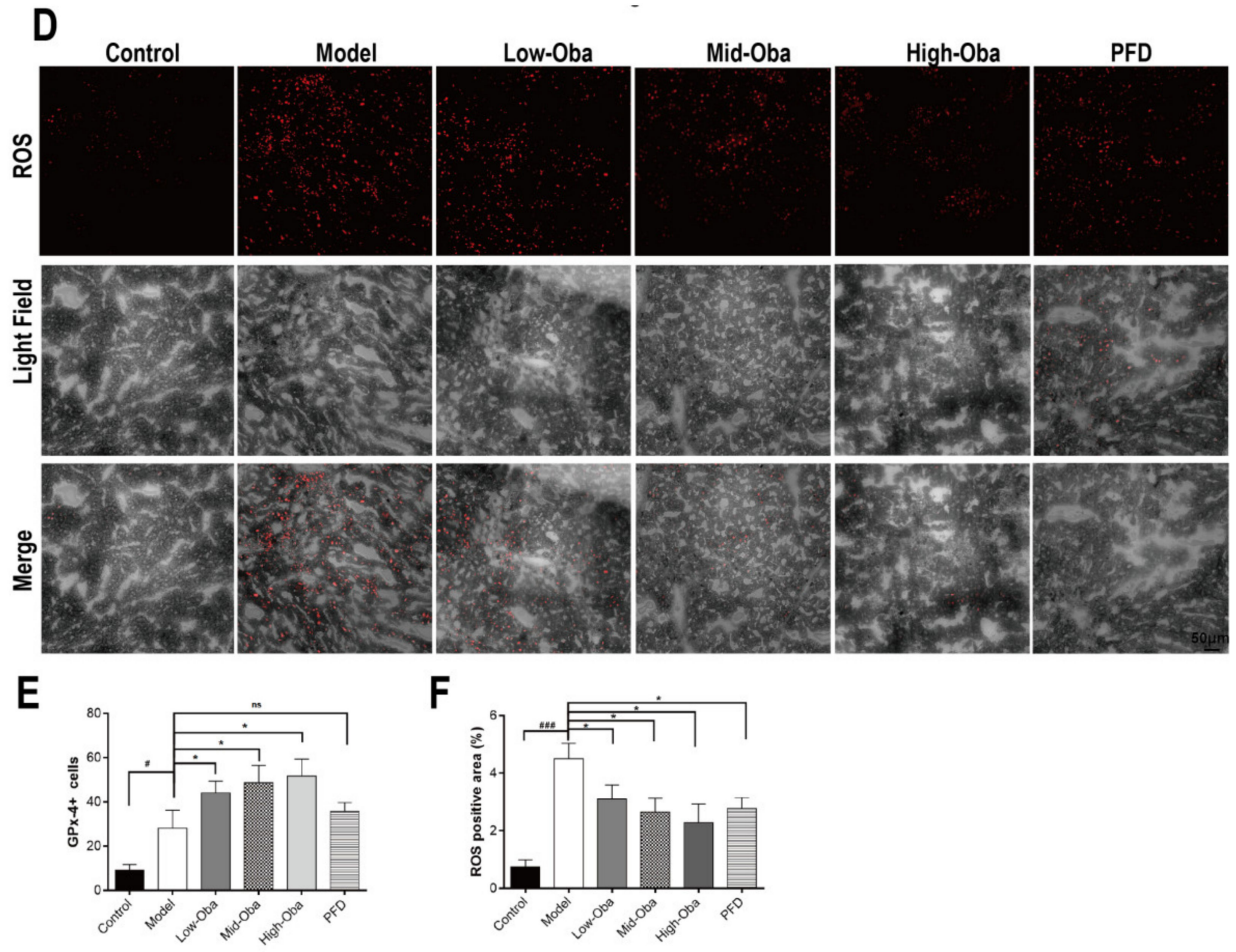
Oba improved the expression of GPx-4, decreased inflammation, and EMT-related protein expression and reduced the lipid oxidation level in CCl4-induced liver fibrosis. (**A**) Quantification of EMT-associated protein and IL-6 expressions as assessed by Western blotting in vivo. (**B**) Quantification of the Nrf-2 and GPx-4 expressions as assessed by Western blotting in vivo. (**C**) Immunohistologic staining for GPx-4 (brown) in the liver tissues. The cell nucleus was counter-stained with DAPI (blue). (**D**) ROS (red) staining by DHE. Tissues morphology of the mouse liver was observed under bright-field microscopy. (**E**) Statistics of the number of GPx-4 positive cells. (**F**) Statistics of ROS positive areas. The results were expressed as means ±SEM (*n* ≥ 3). ns (italic), ^#^, ^##^, ^###^ indicate *p* > 0.05, *p* < 0.05, *p* < 0.01, *p* < 0.001 compare with control group; ns, *, ** indicate *p* > 0.05, *p* < 0.05, *p* < 0.01 compared with model group.

As shown in Figure 5A, Oba inhibited the protein expressions of IL-6 and the occurrence of EMT in mice fibrotic liver with reduced levels of snail and N-cadherin but higher level of E-cadherin. In addition, the protein expressions of Nrf-2 and GPx-4 were obviously increased after treatment with medium and high doses of Oba (Figure 5B). Immunohistochemical Staining (IHC) results demonstrated that, as compared with the model group, the numbers of GPx-4 positive cells (brown) in the high- and medium-dose Oba groups were significantly increased (Figure 5C,E). We next tested the levels of oxidative stress in mice livers and found that, as compared to the control, the model group’s ROS fluorescence intensity was apparently higher. On the other hand, the level of ROS was significantly decreased after treatment with Oba (Figure 5D,F).

## 3. Discussion

Fibrosis is an intimating illness in the world. Whatever the etiology, including virus infection, alcoholic or nonalcoholic hepatitis, and autoimmune responses, all end up with fibrosis due to chronic liver injury [1]. The activation of HSCs is the central event underlying liver fibrosis. HSCs are activated with aberrant productions of a variety of fibrogenic factors and ECM depositions [2,3]. TGF-β related canonical Smad activation signaling, EMT process as well as IL-6 related chronic inflammation are reported to be involved in the above processes [3,4]. In order to evaluate the anti-fibrosis effects of Oba on liver fibrosis, firstly we are focusing on defining its effects on the Lx-2 activation induced by TGF-β, and then verify its in-vivo anti-fibrosis effects by using CCl4-induced liver fibrosis mice model in the end.

Oba is a natural limonoid compound [24] and its biological effects mainly include anti-inflammatory [25], antioxidant [26], and anti-tumor [27] functions. Recently, it is found that Oba is a strong and effective Nrf-2 agonist and exerts anti-pulmonary fibrosis effects in mice [28]. However, the evidence is still lacking in its anti-liver fibrosis effects and related molecular mechanisms. Here, over-expressions of α-SMA, collagen 1, CTGF were all inhibited by Oba in Lx-2 models stimulated with TGF-β. The enhanced EMT signaling is also effectively suppressed by Oba.

It has been confirmed that oxidative stress is an important contributor to fibrosis [12]. Reactive oxygen species (ROS) is an umbrella term for the oxidized molecules or ions with biological activity, and overproductions of various ROS in hepatocytes may lead to its death and elicit liver fibrosis [13]. On the contrary, resting HSCs are activated following liver cell injury and excessive ROS amplifies more HSCs activation and ends up with liver fibrosis [12]. Therefore, dampening the exaggerated oxidative stress becomes an effective treatment for fibrosis.

Nrf-2 is a critical regulator of anti-oxidative stress, the activated phosphorylated Nrf-2 is translocated into the nucleus to drive the up-regulations of the downstream antioxidant proteins, GST, HO1 [15]. Many natural chemical activators of Nrf2 are reported to possess the anti-fibrosis effects, such as tert-butylhydroquinone and curcumin. Among all the anti-oxidant proteins, glutathionperoxidase-4(GPx-4) is a member of the glutathione peroxidase family (GPx1-8) and it facilitates the redox balance via reducing the glutathione [30,31,32]. It is proved that the higher expressions of glutathione peroxidase-7 inhibited non-alcoholic fatty liver disease via decreasing production of ROS [33]. GPx-4 reduced phospholipid peroxide on the lipid membrane to protect cells from oxidative injury [15]. Kazuya Tsubouchi et al. found that the expression of GPx-4 is reduced in the mouse lung fibrosis tissues induced by bleomycin, and the fibrosis is more severe when GPx-4 gene is knocked out. Besides, lipid peroxidation and TGF-β/P-Smad pathway are enhanced significantly in lung fibroblast when GPx-4 gene is depleted [17]. Recently, more evidence supported that phospholipid-derived ROS are involved in special cell death, called ferroptosis. GPx-4 inhibitor, RSL3, is applied as the ferroptosis inducer too [21,34].

In our experiments, we found that Oba exerted the antioxidant capacity by up-regulating Nrf-2 and GPx-4 and down-regulating Nox-2. Notably, the anti-fibrosis effect of Oba was significantly weakened, when GPx-4 gene was inhibited by RSL3, the GPx-4 inhibitor. Consistently, the reduced ROS level by Oba treatment was relightened too. These in vitro observations highly supported that GPx-4-mediated anti-phospholipid ROS were critical for the protective effects of Oba.

In order to validate the above anti-fibrosis treatment results of Oba in LX2 cells, CCl4-induced mice liver fibrosis was examined too. CCl4 is a type of hepatotropic poison, microrosomal enzyme P450 mediated degradation produced highly reactive free radicals to destroy the integrity of cell membrane, in turn, triggered a series of liver dysfunction and injury, eventually evolved into liver fibrosis [35]. In our experiment, Oba displayed good curative effects on CCl4-induced liver fibrosis. It alleviated the liver damage with a lower level of AST, the area of collagen fiber bundles became smaller, and the protein expressions of a-SMA, CTGF, Collagen-1, snai1, N-cadherin, and IL-6 were all reduced. While the anti-oxidant signaling, the protein expressions of Nrf-2, GPx-4 were all elevated too, besides the higher levels of ROS was declined. These results further confirmed the in vitro anti-fibrosis effects by Oba and related signaling, especially inhibition of EMT and anti-oxidant effects by Nrf-2 and GPx-4.

In summary, we concluded that Oba alleviated TGF-β-induced HSCs activation and CCl4-induced hepatic fibrosis through a variety of mechanisms. Firstly, Oba inhibited the classical TGF-β/Smad pathway, EMT, and IL-6 production. Secondly, it displayed excellent antioxidant capacity by enhancing the expressions of Nrf-2 and GPx-4. GPx-4 mediated anti-phospholipid ROS were mainly contributing to its anti-fibrosis effects. Therefore, Oba may serve as a candidate chemical regent for fibrosis prevention and treatment.

Oba showed some curing effect on the CCl4-induced liver fibrosis in C57 mice. Oba could down-regulate the expression of fibrosis protein biomarkers, such as: α-SMA, CTGF, etc. Meanwhile, Oba reduced the protein expression of IL-6, E-cadherin, etc. It was interesting that Mid-Oba show signs of better outcomes over to low-Oba and high-Oba in terms of relative expression of these proteins, although, it is not statistically significant. However, it indicated that the optimum dosage could be a critical factor in this process, and we will explore this phenomenon in the next experiment. Moreover, we found that lower ROS and degree of liver fibrosis with up-regulating expressions of Nrf-2 and GPx-4. So, we believe that Oba could relieve liver fibrosis by regulating the Nrf-2 and GPx-4. However, PFD might have better therapeutic action than Oba both in vivo and vitro. However, Oba was better than PFD on anti-oxidant effect, it is conducive to increase the possibility of finding the new drug for anti-liver-fibrogenic effect from anti-oxidant side, and make a little contribution to human health.

## 4. Materials and Methods

### 4.1. Chemicals and Reagents

Oba (no. B20553, purity ≥ 98%) and RSL3 (no. S81241, purity ≥ 98%) were purchased from YUANYE Bio-Technology Co., Ltd. (Shanghai, China) and dissolved in dimethyl sulfoxide (DMSO) (no. R21950; YUANYE) to a concentration of 72.64 mg/mL and 6 mmol, respectively. After filter sterilization, the resultant solution was preserved at −20 ℃. The solution was added to culture medium to achieve a final DMSO concentration of <1%. TGF-β was purchased from PeproTech China (Shuzou, China). CCl4 was purchased from Jiangsu Qiangsheng Chemical (Jiangsu, China). A ROS fluorescence probe kit was purchased from APPLYGEN (Beijing, China). Trizol, SYBR Premix Ex Taq reagents, RevertAid First Strand cDNA Synthesis Kit, RIPA, BCA Protein Assay kit, and SDS-PAGE kit was purchased from Thermo Fisher Scientific (Waltham, MA, USA). The Cell Counting Kit-8 (CCK8) was purchased from Saint-Bio (Shanghai China). The primers were designed and synthesized by TSINGKE (Xi’an, China). DAPI, Goat Anti-Mouse IgG H&L (Alexa Fluor^®^ 594) (ab150120), anti-alpha smooth muscle actin antibody (ab7817), anti-vimentin antibody (ab20346), anti-IL-6 antibody (ab233706), anti-collagen-1 antibody (ab34710), anti-Smad3 (phospho) antibody (ab52903), and anti-NOX2/gp91phox antibody (ab80508) were purchased from ABcam (Cambridge, MA, USA). E-cadherin polyclonal antibody (20874-1-AP), N-cadherin polyclonal antibody (22018-1-AP), Gpx4 antibody (14432-1-AP), β-actin polyclonal antibody (20536-1-AP), NRF2 polyclonal antibody (16396-1-AP), and KEAP1 polyclonal antibody (10503-2-AP) were purchased from Proteintech (Rosemount, MN, USA). CTGF polyclonal antibody (WL02602) and Snail polyclonal antibody (WL01863) were purchased from Wanleibio (Shenyang, China).

### 4.2. Cell Cultures and Experimental Design

The human hepatic stellate cell line Lx-2 and the human liver cancer cell line Hepg-2 (all sourced from the Department of Pharmacy, The Fourth Military Medical University, China) were cultured in Dulbecco’s Modified Eagle Medium (DMEM) supplemented with 10% fetal bovine serum and 1% penicillin (Thermo Fisher Scientific) and streptomycin (Thermo Fisher Scientific), and then maintained at 37 ℃ in a humidified atmosphere of 5% CO2 and 95% air. To evaluate the effect of Oba on the Lx-2 cell viability, Lx-2 was planked with 1 × 10^4^ cells/well in a 96-well plate and incubated for 24 h before the addition of Oba. Then, Lx-2 were treated with different concentrations of Oba (10, 20, 40, 80, and 160 μM) and cultured for 24, 48, or 72 h. The cell viability was tested by the CCK8. To evaluate the effect of Oba on Lx-2 activation, Lx-2 was pre-cultured with a serum-free medium for 12 h, followed by stimulation with TGF-β (10 ng/mL, 24 h) and treatment with Oba or PFD. To evaluate the effect of Oba on inflammation, Hepg-2 was stimulated by CCl4 (20 nm, 8 h) and treated with 20 μM Oba. At the end of culturing, the cells were collected and subjected to Western blotting and real-time qPCR analysis.

### 4.3. Animals and Experimental Design

Male C57 mouse weighing 20 ± 0.5 g (The Laboratory Animal Center of The Fourth Military Medical University) were housed under appropriate conditions of 25 °C ± 2 °C temperature and 12-h light/dark cycle with free access to water and food. Every experiment in this study was performed in accordance with the protocols approved by the Fourth Military Medical University Committee on Animal Care. The c57 mice were randomly distributed into 6 groups (*n* = 6 in each group) as follow: *i*) sham control group, *ii*) model group, *iii*) high-Oba group (CCl4 + 6 mg/kg Oba), *iv*) medium-Oba group (CCl4 + 3 mg/kg Oba), *v*) low-Oba group (CCl4 + 1.5 mg/kg Oba), and *vi*) PFD group (CCl4 + PFD). The model group was treated with CCl4 (30% CCl4/olive oil, 5 mL/kg intraperitoneal injection) thrice a week for a 6-week period to induce liver fibrosis. The other groups were fed with different doses of Oba or PFD and equal volumes of CCl4 intraperitoneal injection for a 6-week period. Equal volume of olive oil instead of CCl4 was administered to C57 by i.p. as a sham control. Every experiment in this article was performed in accordance with the protocols approved by the Fourth Military Medical University Committee on Animal Care.

### 4.4. Western Blotting

C57 livers or cultured cells were homogenized or lysed in RIPA buffer by using the tissue lapping apparatus or cell scraping (Servicebio, KZ-2). Protein concentration was measured using the BCA Protein Assay Kit. The lysate was centrifuged (4 °C, 12,000 rpm, 5 min), and the supernatant was collected. The operating steps were followed as previously reported [14]. The samples were scanned under a Chemiluminescent Imaging and Analysis system (Beijing Sage Creation Science Co, MiniChemi 610).

### 4.5. Real-Time qPCR Analysis

C57 livers or cultured cells were homogenized or lysed in Trizol reagent with the help of the tissue lapping apparatus to extract total RNA. The total RNA was reverse-transcribed into cDNA by using the RevertAid First Strand cDNA Synthesis Kit. β-actin was used for internal reference. RT-qPCR was performed on the 7500 Real-time qPCR System (Applied Biosystems) with the SYBR Premix Ex Taq reagents. The primers of human genes used were listed in Table 1.

### 4.6. Immunofluorescence Staining and Immunohistochemical Staining (IHC)

The liver tissue sections and LX-2 cell samples were collected for immunofluorescence staining. The samples were incubated with DAPI and primary and secondary antibodies. Next, the samples were scanned under a fluorescence microscope (OlympusU-HGLGPS, Tokyo, Japan).

### 4.7. Biochemical Analyses

The levels of serum alanine transaminase (ALT) and aspartate transaminase (AST) were determined by the Servicebio laboratories (Wuhan, China).

### 4.8. ROS Analyses

The ROS fluorescence probe kit was used to estimate the level of ROS in strict accordance with the instructed specifications. These samples were scanned under the fluorescence microscope.

### 4.9. Histological Assays

The liver tissues were fixed in 4% paraformaldehyde, paraffin-embedded, and sectioned. These sections were then stained with Masson’s trichrome stains to estimate liver fibrosis.

### 4.10. Statistical Analysis

Data were expressed as mean ± SEM. Differences between the groups were determined by a two-tailed Student’s *t* test using the GraphPad Prism 6 software. *p* < 0.05 was considered to be statistically significant.

## Figures and Tables

**Table 1:** The primers of human genes used.

Gene	Forward Primer (5′-3′)	Reverse Primer (5′-3′)
α-SMA	CCGACCGAATGCAGAAGGA	ACAGAGTATTTGCGCTCCGAA
Collagen-1	CCCGGGTTTCAGAGACAACTTC	TCCACATGCTTTATTCCAGCAATC
Vimentin	CGGGAGAAATTGCAGGAGGA	AAGGTCAAGACGTGCCAGAG
IL-6	GAGTAGTGAGGAACAAGCCAGAG	CTACATTTGCCGAAGAGCC
CTGF β-Actin	CTG GCG GCT TAC CGA CTG GAGGCACTCTTCCAGCCTTC	GGC TCT GCT TCT CTA GCC TG GGATGTCCACGTCACACTTC

## Data Availability

Any data can be available from authors.

## Sample Availability

Samples of the compounds are available from the authors.

## References

[1] Wang W., Huang X., Fan X., Yan J., Luan J. (2020). Progress in evaluating the status of hepatitis C infection based on the functional changes of hepatic stellate cells (Review). Mol. Med. Rep..

[2] Lee Y.A., Wallace M.C., Friedman S.L. (2015). Pathobiology of liver fibrosis: A translational success story. Gut.

[3] Walraven M., Hinz B. (2018). Therapeutic approaches to control tissue repair and fibrosis: Extracellular matrix as a game changer. Matrix Biol..

[4] Seki E., Schwabe R.F. (2015). Hepatic inflammation and fibrosis: Functional links and key pathways. Hepatology.

[5] Fontana A., Constam D.B., Frei K., Malipiero U., Pfister H.W. (1992). Modulation of the immune response by transforming growth factor beta. Int. Arch. Allergy Immunol..

[6] Siegel P.M., Massague J. (2003). Cytostatic and apoptotic actions of TGF-beta in homeostasis and cancer. Nat. Rev. Cancer.

[7] Ahmadi A., Najafi M., Farhood B., Mortezaee K. (2019). Transforming growth factor-beta signaling: Tumorigenesis and targeting for cancer therapy. J. Cell. Physiol..

[8] Ansa-Addo E.A., Zhang Y., Yang Y., Hussey G.S., Howley B.V., Salem M., Riesenberg B., Sun S., Rockey D.C., Karvar S. (2017). Membrane-organizing protein moesin controls Treg differentiation and antitumor immunity via TGF-beta signaling. J. Clin. Investig..

[9] Kalluri R., Weinberg R.A. (2009). The basics of epithelial-mesenchymal transition. J. Clin. Investig..

[10] Lu C., Yang Z., Yu D., Lin J., Cai W. (2020). RUNX1 regulates TGF-beta induced migration and EMT in colorectal cancer. Pathol. Res. Pract..

[11] Annaldas S., Saifi M.A., Khurana A., Godugu C. (2019). Nimbolide ameliorates unilateral ureteral obstruction-induced renal fibrosis by inhibition of TGF-beta and EMT/Slug signalling. Mol. Immunol..

[12] Luangmonkong T., Suriguga S., Mutsaers H.A.M., Groothuis G.M.M., Olinga P., Boersema M. (2018). Targeting Oxidative Stress for the Treatment of Liver Fibrosis. Rev. Physiol. Biochem. Pharmacol..

[13] Wan S., Luo F., Huang C., Liu C., Luo Q., Zhu X. (2020). Ursolic acid reverses liver fibrosis by inhibiting interactive NOX4/ROS and RhoA/ROCK1 signalling pathways. Aging.

[14] Gong Y., Yang Y. (2020). Activation of Nrf2/AREs-mediated antioxidant signalling, and suppression of profibrotic TGF-beta1/Smad3 pathway: A promising therapeutic strategy for hepatic fibrosis—A review. Life Sci..

[15] Wang Y., Peng X., Zhang M., Jia Y., Yu B., Tian J. (2020). Revisiting Tumors and the Cardiovascular System: Mechanistic Intersections and Divergences in Ferroptosis. Oxid. Med. Cell. Longev..

[16] Aleksunes L.M., Manautou J.E. (2007). Emerging role of Nrf2 in protecting against hepatic and gastrointestinal disease. Toxicol. Pathol..

[17] Tsubouchi K., Araya J., Yoshida M., Sakamoto T., Koumura T., Minagawa S., Hara H., Hosaka Y., Ichikawa A., Saito N. (2019). Involvement of GPx4-Regulated Lipid Peroxidation in Idiopathic Pulmonary Fibrosis Pathogenesis. J. Immunol..

[18] Wang Y., Li C., Gu J., Chen C., Duanmu J., Miao J., Yao W., Tao J., Tu M., Xiong B. (2020). Celastrol exerts anti-inflammatory effect in liver fibrosis via activation of AMPK-SIRT3 signalling. J. Cell. Mol. Med..

[19] Ahmad A., Ahmad R. (2014). Resveratrol mitigate structural changes and hepatic stellate cell activation in N’-nitrosodimethylamine-induced liver fibrosis via restraining oxidative damage. Chem. Biol. Interact..

[20] Smeuninx B., Boslem E., Febbraio M.A. (2020). Current and Future Treatments in the Fight Against Non-Alcoholic Fatty Liver Disease. Cancers.

[21] Yu Y., Jiang L., Wang H., Shen Z., Cheng Q., Zhang P., Wang J., Wu Q., Fang X., Duan L. (2020). Hepatic transferrin plays a role in systemic iron homeostasis and liver ferroptosis. Blood.

[22] Salah M.M., Ashour A.A., Abdelghany T.M., Abdel-Aziz A.H., Salama S.A. (2019). Pirfenidone alleviates concanavalin A-induced liver fibrosis in mice. Life Sci..

[23] Poo J.L., Torre A., Aguilar-Ramirez J.R., Cruz M., Mejia-Cuan L., Cerda E., Velázquez A., Patiño A., Ramírez-Castillo C., Cisneros L. (2020). Benefits of prolonged-release pirfenidone plus standard of care treatment in patients with advanced liver fibrosis: PROMETEO study. Hepatol. Int..

[24] Luo X., Yue B., Yu Z., Ren Y., Zhang J., Ren J., Wang Z., Dou W. (2020). Obacunone Protects Against Ulcerative Colitis in Mice by Modulating Gut Microbiota, Attenuating TLR4/NF-kappaB Signaling Cascades, and Improving Disrupted Epithelial Barriers. Front. Microbiol..

[25] Gao Y., Hou R., Liu F., Liu H., Fei Q., Han Y., Cai R., Peng C., Qi Y. (2018). Obacunone causes sustained expression of MKP-1 thus inactivating p38 MAPK to suppress pro-inflammatory mediators through intracellular MIF. J. Cell. Biochem..

[26] Zhou J., Wang T., Wang H., Jiang Y., Peng S. (2019). Obacunone attenuates high glucose-induced oxidative damage in NRK-52E cells by inhibiting the activity of GSK-3beta. Biochem. Biophys. Res. Commun..

[27] Murthy K.N., Jayaprakasha G.K., Patil B.S. (2015). Cytotoxicity of obacunone and obacunone glucoside in human prostate cancer cells involves Akt-mediated programmed cell death. Toxicology.

[28] Xu S., Chen W., Xie Q., Xu Y. (2016). Obacunone activates the Nrf2-dependent antioxidant responses. Protein Cell.

[29] Shin D., Kim E.H., Lee J., Roh J.L. (2018). Nrf2 inhibition reverses resistance to GPX4 inhibitor-induced ferroptosis in head and neck cancer. Free Radic. Biol. Med..

[30] Guan Y., Tan Y., Liu W., Yang J., Wang D., Pan D., Sun Y., Zheng C. (2018). NF-E2-Related Factor 2 Suppresses Intestinal Fibrosis by Inhibiting Reactive Oxygen Species-Dependent TGF-beta1/SMADs Pathway. Dig. Dis. Sci..

[31] Wang Z., Chen Z., Li B., Zhang B., Du Y., Liu Y., He Y., Chen X. (2020). Curcumin attenuates renal interstitial fibrosis of obstructive nephropathy by suppressing epithelial-mesenchymal transition through inhibition of the TLR4/NF-kB and PI3K/AKT signalling pathways. Pharm. Biol..

[32] Toppo S., Flohe L., Ursini F., Vanin S., Maiorino M. (2009). Catalytic mechanisms and specificities of glutathione peroxidases: Variations of a basic scheme. Biochim. Biophys. Acta.

[33] Kim H.J., Lee Y., Fang S., Kim W., Kim H.J., Kim J.W. (2020). GPx7 ameliorates non-alcoholic steatohepatitis by regulating oxidative stress. BMB Rep..

[34] Ousingsawat J., Schreiber R., Kunzelmann K. (2019). TMEM16F/Anoctamin 6 in Ferroptotic Cell Death. Cancers.

[35] Weber L.W., Boll M., Stampfl A. (2003). Hepatotoxicity and mechanism of action of haloalkanes: Carbon tetrachloride as a toxicological model. Crit. Rev. Toxicol..

